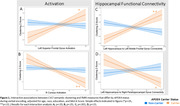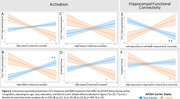# APOE4‐related brain vulnerabilities in memory circuitry of midlife women

**DOI:** 10.1002/alz.092813

**Published:** 2025-01-09

**Authors:** Katrina A Wugalter, Rebecca C Thurston, Rachel A Schroeder, Minjie Wu, Howard J Aizenstein, M. Ilyas Kamboh, Carol A. Derby, Pauline M Maki

**Affiliations:** ^1^ University of Illinois Chicago, Chicago, IL USA; ^2^ Department of Psychiatry, School of Medicine, University of Pittsburgh, Pittsburgh, PA USA; ^3^ Department of Bioengineering, University of Pittsburgh, Pittsburgh, PA USA; ^4^ University of Pittsburgh Alzheimer's Disease Research Center, Pittsburgh, PA USA; ^5^ University of Pittsburgh Alzheimer's Disease Research Center (ADRC), Pittsburgh, PA USA; ^6^ University of Pittsburgh, Pittsburgh, PA USA; ^7^ Department of Neurology, and Department of Epidemiology and Population Health, Albert Einstein College of Medicine, Bronx, NY USA; ^8^ Department of Psychiatry, University of Illinois at Chicago, Chicago, IL USA

## Abstract

**Background:**

The APOE4 genotype appears to confer differential risk of Alzheimer’s disease for women compared to men. As APOE4 effects in midlife women can be subtle (e.g., negligible main effects of APOE4 genotype on cognition and brain outcomes), there is a need for early markers of APOE4‐related brain vulnerabilities to identify those at early risk and guide treatment. We therefore examined whether patterns of brain activity and hippocampal functional connectivity that support verbal memory differ by APOE4 status in midlife women participating in MsBrain, a cohort study of brain health in midlife women.

**Method:**

Participants completed functional magnetic resonance imaging (fMRI) assessments during verbal encoding and recognition tasks. Generalized psychophysiological interaction (gPPI) analyses estimated hippocampal functional connectivity using SPM12 and Conn, with AlphaSim (AFNI) to correct for multiple comparisons. Associations between CVLT indices and brain outcomes (regional activation, left [LHipp] and right hippocampal [RHipp] functional connectivity) were tested via multiple linear regression controlling for age, education, race, and MoCA score. Interactions by APOE4 status (carriers [E3E4 and E4E4] versus non‐carriers [E3E3]) were tested.

**Result:**

In 166 women (mean age=59.31 years, 83.7% white, 32% APOE4 carriers), there was no main effect of APOE4 on verbal memory performance, activation, or hippocampal connectivity. During encoding, APOE4 status moderated the associations between semantic clustering and fMRI measures (e.g., activation of the left superior frontal gyrus and connectivity from LHipp to left middle frontal gyrus and to right parahippocampal gyrus; Figure 1). These associations during encoding were stronger in APOE4 carriers versus noncarriers. During recognition, APOE4 status moderated the associations between: a) total learning and activation of right inferior, middle, and superior frontal gyri; b) clustering and activation of the right superior frontal gyrus; c) total learning and LHipp connectivity to left middle temporal gyrus; and d) clustering and RHipp connectivity to right superior temporal gyrus (Figure 2). For recognition, APOE4 carriers typically showed decreased activation/connectivity, while noncarriers showed neutral or enhanced activation/connectivity.

**Conclusion:**

These findings may indicate early compensatory mechanisms in female midlife APOE4 carriers that support verbal memory, serving as potential markers of risk and targets for intervention in midlife APOE4 carriers.